# Current Topics in Gamma-Ray Astrophysics

**DOI:** 10.6028/jres.105.012

**Published:** 2000-02-01

**Authors:** Grant J. Mathews, P. Maronetti, Jay Salmonson, J. R. Wilson

**Affiliations:** University of Notre Dame, Department of Physics 225 Nieuwland Science Hall, Notre Dame, IN 46556; Lawrence Livermore National Laboratory, Livermore, CA 94550

**Keywords:** gamma-ray bursts, neutrino bursts, supernovae

## Abstract

This paper reports on recent progress toward unraveling the origin of gamma-ray bursts. It is concluded that neutron-star binaries are one of the few remaining candidates. A model is proposed based upon general relativistic hydrodynamic studies which indicate a new physical process by which to power a gamma-ray burst. Relativistically driven compression, heating, and collapse of the individual neutron stars can occur many seconds before inspiral and merger. This compression may produce a neutrino burst of ∼10^53^ ergs lasting several seconds. The associated thermal neutrino emission produces an *e*^+^–*e* ^−^ pair plasma by $v\bar{v}$ annihilation. We show first results of a simulated burst which produces ∼10^51^ erg in γ rays of the correct spectral and temporal properties.

## 1. Introduction

There are numerous aspects of modern gamma-ray astrophysics which are intimately connected with high precision gamma-ray astronomy. High resolution observations of galactic gamma-ray lines have provided profound insight into the internal workings of supernovae as well as their recent history in the Galaxy. Since this topic is considered in another paper from this conference, we concentrate here on the other exciting topic in current gamma-ray astrophysics—gamma-ray bursts. Indeed, the papers from this conference are inextricably immersed in efforts to detect emitters of gamma radiation. For that reason, discussion of gamma-ray detection ought to include a discussion of the ultimate emitter of gamma-radiation in the universe—the mysterious originator of gamma-ray bursts (GRBs). The puzzle of the origin of gamma-ray bursts has been with us since they were first discovered [1] in the 1970s. Although they appear to strike earth daily from an isotropic distribution in the sky [2]. They exhibit no confirmed gamma-ray lines in a spectrum that is not obviously thermal. Attempts to identify their source have not met with success—at least until recently.

The problem has certainly not been one of limited theories. Well over 100 papers have been written attempting to explain GRB origin [3]. The real problem has been a failure to find an optical counterpart to identify the source location. This was due to the limited angular resolution of GRB detectors which permitted thousands of possible sources within any given error box. The NASA Compton Gamma-Ray Observatory, for example, though identifying thousands of bursts [2], has been able to locate only a handful of bursts [4] to better than 1° precision, and none to the minute resolution required to find an optical counterpart.

This uncertainty has led to two proposed sites for GRB origin: 1) galactic (∼10 kpc to 300 kpc) sources with an implied 4π gamma-ray burst energy of 10^37^ erg to 10^39^ erg; or 2) a cosmological (>100 Mpc) source with an energy of ∼10^51^ erg to 10^52^ erg. Opinion as to which source is most implied by the data has remained divided into two camps, until recently.

The field has changed in the past year, however, due to high-resolution burst detections from the BeppoSax X-Ray satellite as well as observations from ASCA, RXTE, and ROSAT. For the first time arcminute gamma-ray burst locations have been determined quickly enough to allow follow-up searches for optical or radio counterparts. These searches have revealed that at least some γ-ray bursts involve weak x-ray, optical, or radio transients, and are of cosmological origin [5]. The Mg I absorption and [O II] emission lines along the line of sight from the GRB970508 optical transient imply [6] a redshift *Z* ≥ 0.835. The implied distance of greater than a 10 billion light years means that this burst must entail a release of ⪞10^51^ erg in γ rays on a time scale of seconds. This requirement has been rendered even more demanding by another recent event [7] in which a GRB appears centered on a galaxy at redshift 3.42. This implies that the energy of a 4π burst would have to be as much as 3×10^53^ erg, comparable to the visible light output of ≈10^9^ galaxies, roughly the entire visible universe!!

Clearly, it is a challenge to explain such an unfathomable burst of energy, and even more perplexing to put essentially all of that energy in nothing but γ rays!! Based upon the accumulated observations we can probably conclude that the following four features characterize the source environment: 1) The ratio of burst energy to the beam solid angle 4π *E/* Ω is ∼10^53^ erg, e.g., with 1 % beaming the burst energy is ≈10^51^ erg; 2) The multiple peak temporal structure of most bursts probably requires multiple colliding shocks [8]; 3) The observed afterglows imply some surrounding material on a scale of light hours; and 4) the presence of OII emission lines suggests that the bursts occur in a young stellar population.

Possible sources consistent with these conditions probably involve some kind of catastrophic collapse/accretion in an environment somewhat depleted in baryons. Some proposed sites include accretion onto supermassive black holes, AGNs, relativistic stellar collisions, hypernovae, and binary neutron star coalescence. Each of these possibilities, however, remains speculative until definitive multi-dimensional models can be constructed for their evolution. For the remainder of this paper we shall discuss a preliminary attempt to construct such a model for relativistically driven GRBs from neutron-star binaries.

It has been speculated for some time that inspiraling neutron stars could provide a power source for cosmo logical gamma-ray bursts. The rate of neutron star mergers (when integrated over the number of galaxies out to high redshift) could account for the observed GRB event rate. Previous, Newtonian and post Newtonian studies [9] of the direct merger of two neutron stars have found that the neutrino emission time scales are so short that it would be difficult to drive a gamma-ray burst from this source. However, our numerical studies of the strong field relativistic hydrodynamics of close neutron star binaries in three spatial dimensions [10,11,12,13] have shown that neutron stars in a close binary can experience relativistic compression and heating over a period of seconds. This effect can cause each of the stars to collapse to two black holes prior to merger. During the compression phase as much as 10^53^ erg in neutrinos can be emitted before the stars collapse [12]. This effect may provide a new mechanism to power cosmological gamma-ray bursts and their x-ray and optical counterparts. Here, we report on preliminary efforts to better quantify this release of neutrino energy around the binary and numerically explore its consequences for the development of a *e*^+^–*e*^–^plasma and associated GRB.

In previous work [12] we computed properties of equal-mass neutron star binaries as a function of mass and EOS. From these we deduced that compression, heating and collapse can occur at times from a few seconds to a few hours before binary merger. Our calculation of the rates of released binding energy and neutron star cooling suggests that interior temperatures as hot as 70 MeV are possible. This leads to several seconds of high neutrino luminosity, *L_v_*≈10^53^ erg s^–1^. This much neutrino luminosity would convert to an *e*^+^–*e*^–^ pair plasma above the stars as is also observed in supernova simulations[14]. This plasma is a viable candidate source for cosmological gamma-ray bursts.

We have studied the transport of this neutrino flux above the neutron star using a modified version of the supernova code of [14]. We find entropies as high as *S*/*k* ≈10^6^ (i.e., few baryons) in the pair plasma above the stars. We have also made a spherical calculation of the hydrodynamic evolution of the pair plasma based upon our calculated neutrino emission and an efficiency (1 % to 10 %) for the conversion of neutrinos to *e*^+^–*e*^–^ pairs. The results are quite encouraging. We inject the pair plasma into a spherical grid at a rate consistent with the compression-induced thermal neutrino emission which itself is determined by the gravitational wave emission time [12]. The plasma is evolved hydrodynamically until it becomes optically thin and the escape of γ rays is calculated. By this time the average temperature is about 10 eV, but the special relativistic gamma-factor is ≈3×10^4^. This produces an integrated photon energy spectrum which is quite typical of observed bursts. It peaks at around 200 keV and extends to a few MeV. Nearly all energy deposited into *e*^+^–*e*^–^ pairs ends up as γ rays.

**Fig. 1:**
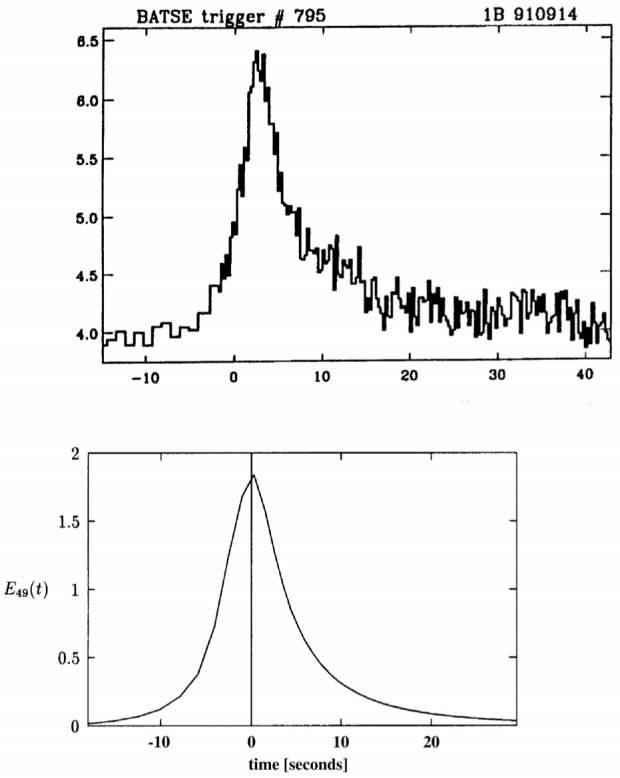
Calculated gamma-ray burst luminosity (lower curve) compared with a similar single burst from the BATSE catalog. The total released energy from the calculated burst is ≈ 10^51^ erg.

Figure 1 shows a calculation of γ-ray burst luminosity as a function of time compared with a typical “single-burst” from the BATSE catalog. The integrated energy in gamma-rays from the calculated burst is ∼ 10^51^ erg. The similarity between the observed and calculated burst is remarkable considering that there has been no parameter fitting in these calculations. Single-burst durations in the model vary from ≈ 1 s to 10 s. We also find that if the masses of the stars differ by more than ∼ 5 % that the γ -ray emission separates into two bursts spaced a number of seconds apart. Indeed, there are a numerous bursts in the BATSE catalog consistent with this morphology. The bimodal character of the burst durations (the so-called t90 distribution) arises naturally in this model from the likelihood that for many bursts only one collapse is observed.

The multiple shock mechanism necessary to account for the typical multiple peak structure observed in many bursts may result from the coupling of the plasma (and jet) evolution to the orbit dynamics. The coupling of magnetic field lines to the fluid motion may also have an effect. We are currently investigating both possibilities. Regarding the magnetic field, we have noted [13] that the neutron-star fluid seems to relax to a nearly spinless state prior to collapse. This spinless state, however acquires net fluid motions and reconnecting field lines relative to the corotating local inertial frame of the binary. These reconnections can cause the magnetic field to grow within the stars.

We have made a preliminary simulation of the growth rate of the magnetic field by introducing an electromagnetic vector potential into the evolution equations. We followed the evolution of the vector potential for 10 ms (about one orbit) assuming that the fluid is a perfect conductor. The magnetic field energy grows exponentially with an e-folding time of about 1 ms. Thus, the field could build up very quickly to a magnitude such that reconnection and back reaction of the fluid inhibits further growth. The limiting fields could approach an equipartition limit as high as 10^17^ gauss just before collapse. As the magneitic field grows it should bubble from the surface. We speculate that interactions of these magnetic bubbles the surrounding pair plasma might lead to the multiple peak structure observed in many GRBs.

Of some relevance to this workshop is the fact that during the neutrino emission phase a small fraction of the surface material is ablated from the stars in a baryon wind not unlike that expected to occur in supernovae [15]. We have estimated that in that case some unique gamma-ray lines will be emitted. The reason is that the baryonic material is likely to evolve from a dissociated neutron-rich gas into heavy nuclei far on the neutron rich side of stability. The material is likely to become optically thin when it is still composed of nuclei far from stability. The decay back to stability should include spectral lines of neutron-rich nuclei for several minutes after the burst. The level of this activity, however, is a small fraction of the bulk of the burst. Hence, the ultimate confirmation of this GRB paradigm will ultimately require the application of extreme high-resolution gamma-ray spectroscopy to test this prediction.
